# Infarct size by SS-SSFP vs. IR-GRE: influence of imaging time after contrast administration and infarct age, and implications for clinical trials

**DOI:** 10.1186/1532-429X-16-S1-P183

**Published:** 2014-01-16

**Authors:** Elisa McAlindon, Chris Lawton, Nauman Ahmed, Nathan Manghat, Mark Hamilton, Chiara Bucciarelli-Ducci

**Affiliations:** 1 CMR Unit, NIHR Bristol Cardiovascular Biomedical Research Unit, Bristol, UK

## Background

Myocardial late gadolinium enhancement (LGE) imaging is conventionally acquired using a gradient-echo inversion recovery (IR-GRE) sequence 15-20 min after contrast administration. However, this method can be limited by poor breath holding or arrhythmias. Free-breathing single shot steady state free precession (SS-SSFP) sequence is an alternative LGE imaging technique which can overcome some of the IR-GRE limitations but at the expense of lower resolution. The ideal imaging timing for LGE SS-SSFP, and whether it can be used interchangeably with IR-GRE has not yet been established. The aim of our study was to investigate acute and chronic infarct size: 1) Comparing LGE by SS-SSFP vs. IR-GRE at 15 min. 2) Comparing LGE SS-SSFP imaging at 5 vs. 10 vs. 15 min after contrast administration vs. IR-GRE at 15 min.

## Methods

36 patients were prospectively recruited for CMR day 2 and 3 months following reperfused STEMI. IR-GRE images were acquired 15 minutes following gadolinium contrast administration (Gadovist 0.1 mmol/kg). Free-breathing SS-SSFP images were obtained 5, 10 and 15 min following contrast. LGE was calculated and infarct size was expressed in grams. Agreement between the 2 sequences was assessed by the Bland Altman method. Differences between time following contrast for both acute and chronic infarct size were assessed using paired t-tests. All patients provided informed written consent and the study was approved by the regional ethics committee.

## Results

When imaging 15 min after contrast administration, there was a significant difference in acute infarct mass using SS-SSFP vs. GRE-IR (p = 0.006), whilst there was no difference in chronic infarct mass (p = 0.54) (Table 1). Bland Altman agreement with IR-GRE was best for chronic infarct (chronic infarct bias -0.81 g, acute infarct bias -7.08 g). When imaging using SS-SSFP in acute infarct at multiple time points, there was no difference between imaging at 5 or 10 min (p = 0.13), but there was a significant difference between 10 and 15 min (p = 0.02) and 5 and 15 min (p = 0.01) (Table 2). When imaging LGE with SS-SSFP in chronic infarct at multiple time points, the only significant difference was noted between 5 and 15 min (p = 0.04) (Table 2).

**Table 1:** LGE Imaging by SS-SSFP vs. IR-GRE 15 min after contrast administration.

LGE Imaging	SS-SSFP 15 min	IR-GRE 15 min	p value
Acute Infarct	28.65 g	35.7 g	p = 0.006

Chronic Infarct	16.3 g	15.5 g	p = 0.54

**Table 2:** LGE Imaging by SS-SSFP at multiple time points after contrast administration.

	5 min	10 min	15 min	p value
Acute Infarct	32.8 g	31.1 g	28.6 g	5 vs 10 min p = 0.13, 10 vs 15 min p = 0.02, 5 vs 15 min p = 0.01

Chronic Infarct	18.6 g	17.4 g	16.3 g	5 vs 10 min p = 0.09, 10 vs 15 min p = 0.35, 5 vs 15 min p = 0.04

## Conclusions

Our study demonstrates both acute and chronic infarct size with SS-SSFP changes significantly between 5 vs. 10 vs. 15 min, demonstrating that the time of imaging after contrast administration is important for this sequence too. The ideal timing for imaging acute and chronic infarct size by SS-SSFP and IR-GRE are different. In particular, when imaging at 15 min after contrast, LGE by SS-SSFP can underestimate acute infarct size. In chronic infarctions time after contrast injection for LGE by SS-SSFP seems less critical but infarct size at 15 min best correlated with LGE by IR-GRE. LGE by SS-SSP offers a valid alternative in clinical practice for the (visual) assessment of myocardial infarction but in clinical trials it should not be used interchangeably with LGE IR-GRE

## Funding

This work was funded by the NIHR Bristol Cardiovascular Biomedical Research Unit.

